# N-terminal lid swapping contributes to the substrate specificity and activity of thermophilic lipase TrLipE

**DOI:** 10.3389/fmicb.2023.1193955

**Published:** 2023-06-26

**Authors:** Yakun Fang, Fan Liu, Yi Shi, Ting Yang, Yu Xin, Zhenghua Gu, Guiyang Shi, Liang Zhang

**Affiliations:** ^1^National Engineering Research Center for Cereal Fermentation and Food Biomanufacturing, Jiangnan University, Wuxi, Jiangsu, China; ^2^Jiangsu Provincial Engineering Research Center for Bioactive Product Processing, Jiangnan University, Wuxi, Jiangsu, China; ^3^Wuxi Food Safety Inspection and Test Center, Technology Innovation Center of Special Food for State Market Regulation, Wuxi, Jiangsu, China

**Keywords:** binding energy, lid swapping, thermophilic lipase, molecular dynamics, substrate specificity

## Abstract

TrLipE is a thermophilic lipase that has potential commercial applications because of its catalytic ability under extreme conditions. Consistent with most lipases, the lid of TrLipE is located over the catalytic pocket, controls the substrate channel to the active center, and regulates the substrate specificity, activity, and stability of the enzyme through conformational changes. TrLipE from *Thermomicrobium roseum* has potential industrial applications, which is hindered by its weak enzymatic activity. Here, 18 chimeras (TrL1-TrL18) were reconstructed by N-terminal lid swapping between TrLipE and structurally similar enzymes. The results showed that the chimeras had a similar pH range and optimum pH as wild TrLipE but a narrower temperature range of 40–80°C, and TrL17 and the other chimeras showed lower optimum temperatures of 70°C and 60°C, respectively. In addition, the half-lives of the chimeras were lower than those of TrLipE under optimum temperature conditions. Molecular dynamics simulations indicated that chimeras had high RMSD, RMSF, and B-factor values. When p-nitrophenol esters with different chains were used as substrates, compared with TrLipE, most of the chimeras had a low *K*_*m*_ and high *k*_*cat*_ value. The chimeras TrL2, TrL3, TrL17, and TrL18 could specifically catalyze the substrate 4-nitrophenyl benzoate, with TrL17 showing the highest *k*_*cat*_/*K*_*m*_ value of 363.88 ± 15.83 L⋅min^–1^⋅mmol^–1^. Mutants were then designed by investigating the binding free energies of TrL17 and 4-nitrophenyl benzoate. The results indicated that single, double, and triple substitution variants (M89W and I206N; E33W/I206M and M89W/I206M; and M89W/I206M/L21I and M89W/I206N/L21I, respectively) presented approximately 2- to 3-fold faster catalysis of 4-nitrophenyl benzoate than the wild TrL17. Our observations will facilitate the development of the properties and industrial applications of TrLipE.

## 1. Introduction

Lipases (EC 3.1.1.3) are well-known biotechnologically relevant biocatalysts of the α/β-hydrolase superfamily that act on carboxylic ester bonds and catalyze a variety of chemical reactions, including hydrolysis, transesterification, esterification, alcoholysis, aminolysis, and acidolysis, in aqueous and organic solvents (Sarmah et al., 2017; Casas-Godoy et al., 2018; Vivek et al., 2022). Based on their thermostability, organic solvent tolerance, and excellent catalytic ability under extreme conditions, lipases have been widely used in the food (Asmat et al., 2019; Cipolatti et al., 2020), detergent, leather (Verma et al., 2021), pharmaceutical, and biodiesel industries (Cavalcante et al., 2020; Zhong et al., 2020; Almeida et al., 2021). Lipases have a highly conserved catalytic triad consisting of nucleophilic serine or cysteine, aspartic or glutamic acid, and histidine (Anobom et al., 2014; Gutiérrez-Domínguez et al., 2022; Liu et al., 2022). Most lipases display a very similar fold in which mixed β-sheets are surrounded by α-helices and a domain named lid that controls the substrate channel to the active pocket (Mohd Din et al., 2021; Xing et al., 2021; Cui et al., 2022).

Most lipases are widely considered to exert interfacial activation, and the lid domain located above the catalytic pocket is responsible for this phenomenon because the lid switches between open and closed conformations at the interface to correspond to the active and inactive states of the lipase, respectively (Jan et al., 2017; Maiangwa et al., 2017; Soni et al., 2019; Wang et al., 2021). However, CALB from *Candida antarctica* with a small lid has also been reported to display almost no interfacial activation in the presence of short-chain substrates (Stauch et al., 2015). The lid plays an important role in most lipases because it not only regulates the activity of the enzyme but also contributes to its catalytic specificity, thermostability, and binding of the substrate. In recent years, considerable research interest has focused on the role of lids in enzymes. Tang et al. (2015) reported that the T66L/D70N mutant of lipase (PEL) from *Penicillium expansum* displayed a 136.4-fold increase in p-nitrophenol palmitate activity by replacing the amino acids of the N-terminal hinge of the lid. Soni et al. (2019) engineered a monoglyceride lipase (TON-LPL) from *Thermococcus onnurineus* into triglyceride lipase by lid swapping. The values of t_1/2_ at 60°C and Tm were increased by introducing a disulfide bridge into the lid hinge of a lipase from *Rhizopus chinensis* (Yu et al., 2012).

The optimal enzyme-substrate complex exhibits good binding affinity and a relatively low binding free energy (Anuar et al., 2021). Therefore, a variety of computational programs have been developed to calculate the binding energy of protein and ligand complexes as a protein mutation design strategy to improve enzyme catalytic efficiency and stereoselectivity (Kumari and Gupta, 2013; Zhang et al., 2013; Bai et al., 2022).

TrLipE from *T. roseum* possesses remarkable enzymatic properties, such as excellent thermostability, organic solvent tolerance, and pH tolerance; thus, it has great potential for industrial application. However, its weak enzymatic activity hinders the application of TrLipE in the above-mentioned fields. In this study, 18 chimeras were successfully constructed by lid swapping between TrLipE and structurally similar enzymes. Different from other studies on lid, our study changed the binding ability of chimeras to different substrates by exchanging the complete lid domains, and thus changing their specificity. In addition, to further improve the enzymatic activity of the chimera, mutation sites were designed based on strategies to reduce the binding energy of the protein–ligand docking complex. This study provides valuable insights into the rational design of TrLipE for further industrial applications.

## 2. Materials and methods

### 2.1. Strains, plasmids, and chemicals

*Escherichia coli* JM109 and BL21 were used for cloning and TrLipE expression, respectively, and stored at −80°C in our laboratory. The ClonExpress II One-Step Cloning Kit was purchased from Vazyme Biotech Co., Ltd. (Nanjing, China). The substrates (p-nitrophenyl fatty acid esters) were purchased from Sigma-Aldrich (USA). A BCA protein quantification kit was purchased from Tiangen Biotech Co., Ltd. (Beijing, China). A Mag-Beads His-Tag Protein Purification Kit was purchased from Sangon Biotech (Shanghai, China). Other analytical reagents and biological materials were provided by local suppliers.

### 2.2. Site-directed mutagenesis, cloning, expression, and purification of TrLipE, chimeras, and mutants

Standard manipulation techniques have been used for the construction and isolation of plasmid DNA and transformation of *E. coli* (Kay et al., 2016). Briefly, the DNA fragment containing the TrLipE gene sequence without a lid and the sequence of plasmid pTIG, and other 18 lid sequences fragments from 18 different lipases similar to TrLipE were obtained by polymerase chain reaction (PCR) using the primers sequence in Supplementary Table 1. Then, the expression plasmids for the chimeras were obtained by homologous recombination of the above fragments, and the expression plasmids for the chimeras were then transformed in *E. coli* JM109 for amplification and preservation. In addition, the primers in Supplementary Table 2 were designed on the recombinant plasmid pTIG-TrL17 for site-directed mutagenesis, and a Mut Express MultiS Fast Mutagenesis Kit V2 (Vazyme) was used for the designed substitutions according to the manufacturer’s recommendations. After PCR, the mixture was digested with *Dpn*I to remove the template (pTIG-TrL17). The plasmids containing mutated sites were then transformed in *E. coli* JM109 for amplification and preservation. After verifying the sequence, the chimeric and mutant expression plasmids were transformed into *E. coli* BL21. Recombinant cells were cultured, harvested, and disrupted by sonication where the program was that the power was set at 39%, work for 1 s, stop for 2 s, and the total time was 15 min. The Mag-Beads His-Tag Protein Purification kit was used for purification of TrLipE, chimeras, and mutants, as described by Fang et al. (2021), and then the pure enzyme was centrifuged (4,000 rpm 30 min) by ultrafiltration to remove imidazole, and the buffer was replaced with Tris-HCL (50 mmol⋅L^–1^, pH 8.0).

### 2.3. Enzyme and protein assays

The TrLipE and chimera enzymatic activity was determined using emulsified p-nitrophenol esters as the substrates (Sha et al., 2013; Fang et al., 2022). The substrates were dissolved in dimethyl sulfoxide at a concentration of 15 mmol⋅L^–1^, and the buffer was composed of 50 mM Tris-HCl containing 0.55 g⋅L^–1^ Arabic gum (sigma) and 1.2 g⋅L^–1^ sodium deoxycholate (sigma) (pH 8.0). The reaction was processed in a final volume of 500 μL, which contained 20 μL enzyme at a concentration of 0.3 mg/mL and 480 μL of substrate mixture, in which the substrates were mixed with the buffer at a ratio of 1:100. The absorbance value at OD_410nm_ was determined. Mixture without the enzyme was applied as the blank. One enzyme unit (μmol⋅mg^–1^⋅min^–1^) was defined as the amount of enzyme that released 1 μmol of p-nitrophenol per minute under the assay conditions. All results were obtained from experiments performed in triplicate, and the variance was calculated.

### 2.4. Effect of temperature and pH on enzyme activity and chimera half-life

Following the enzyme activity assay, the optimum temperature and pH were determined within a temperature range of 40–80°C and a pH range of 5.5–11 using buffers with different pH values, including phosphate buffer (pH = 5.5–7.5), Tris-HCl buffer (pH = 7.5–9.0) and glycine-sodium hydroxide buffer (pH = 9.0–11.0). The maximum enzymatic activity was defined as the control (100%).

For determining the half-life, the pure enzyme solution was placed at constant temperatures of 60, 70, and 80°C for TrL17 and 50, 60, and 70°C for the other chimeras, and then samples were collected at intervals of 1 h for chimeras and 3 h for TrLipE to determine the remaining enzyme activity as described above. As mentioned above, enzymatic activity at 0 h was defined as the control (100%).

### 2.5. Substrate specificity and kinetic parameters

Substrate specificity was determined using different p-nitrophenyl esters, including p-NP acetate (C2), p-NP butyrate (C4), p-NP hexanoate (C6), p-NP caprylate (C8), p-NP decanoate (C10), p-NP laurate (C12), p-NP palmitate (C16), and p-NP benzoate. The *K*_*m*_ value was calculated using the Non-linear Curve Fit, and the *k*_*cat*_ and *k*_*cat*_/*K*_*m*_ values were calculated according to the molecular weight and concentration of the enzyme. For each substrate, the reaction was determined at 60°C. All results were obtained from experiments performed in triplicate, and the variance was calculated.

### 2.6. Computational methods

After modeling with Alphafold2, molecular dynamics (MD) simulations of chimeras and TrLipE were performed as described by Fang et al. (2021), Kay et al. (2016). Briefly, the Gromacs 2018.4 package (Tambunan et al., 2014) containing the Amber14SB (Maier et al., 2015) all-atom force field was used under constant temperature, pressure, and periodic boundary conditions. MD runs were performed for 100 ns with a time step of 2 fs at 300 K. The molecular docking of TrL17 with 4-nitrophenyl benzoate was carried out using Schrödinger software (Godara et al., 2022), and the docking results were converted into PDB format using PyMOL. Discovery Studio 2019b (DS) was used to analyze the docking and virtual mutations (Gao et al., 2018).

## 3. Results and discussion

### 3.1. Enzyme preparation of chimeras and TrLipE

The amino acid sequence of TrLipE was submitted to the PDB database for alignment, and 18 crystal structures with 20–60% similarity to TrLipE were selected to reconstruct chimeras. Table 1 shows the lid sequence range used to swap, the PDB code of these 18 crystal structures, and the names of the chimeric enzymes correspond to different lids. After recombination between TrLipE and the lids of the above-mentioned structures, the gene sequences were optimized and synthesized *E. coli*. After expression and purification according to the above method, the pure enzyme was further verified by SDS-PAGE (12% separating gel). The results indicated that these chimeras could be expressed efficiently in *E. coli* BL21 and showed a band with the calculated molecular mass range of approximately 35–37 kDa because of the different sizes of the lid regions. Furthermore, the chimeras displayed different expression levels than TrLipE. The reason for this phenomenon was that the proportion of hydrophilic amino acids in the lid region changed after lid swapping (Supplementary Table 3).

**TABLE 1:** The PDB code of 18 crystal structures, lid sequences range used to swap and the names of chimeric enzymes correspond to different lid.

PDB code	Residues of lid for chimeras	Residues of TrLipE for chimeras	Name of chimeras
1EVQ (De Simone et al., 2000)	Leu3-Pro44	Val54-Ala323	TrL1
3H18 (Nam et al., 2009)	Ala2-Asp42		TrL2
3K6K (Nam et al., 2010)	Asp13-Gly57		TrL3
3QH4 (McKary et al., 2015)	Val2-Gly53		TrL4
4C89 (Alvarez et al., 2014)	Asn23-Pro66		TrL5
4N5H	Ala2-Asp34		TrL6
4P9N (Ohara et al., 2014)	Pro2-Lys45		TrL7
4V2I (De Santi et al., 2016)	Pro2-Asp49		TrL8
4YPV (Pereira et al., 2017)	Ala2-Gly49		TrL9
4ZRS (Cao et al., 2015)	Thr2-glu38		TrL10
5HC0 (Huang et al., 2016)	Lys4-Gly69		TrL11
5MIF (Cavazzini et al., 2017)	Gln34-Glu74		TrL12
6AAE (Kim et al., 2019)	Pro2-Gln42		TrL13
6K34	Arg8-Arg52		TrL14
6RJ8 (Caputi et al., 2020)	Asp9-Gly46		TrL15
7B1X (Boyko et al., 2021)	Asn2-Cys49		TrL16
7B4Q (Noby et al., 2021)	Lys2-Glu45		TrL17
7UAY (Denison et al., 2022)	Ala2-Glu45		TrL18

### 3.2. Characterization of TrLipE and chimeras

**FIGURE 1:**
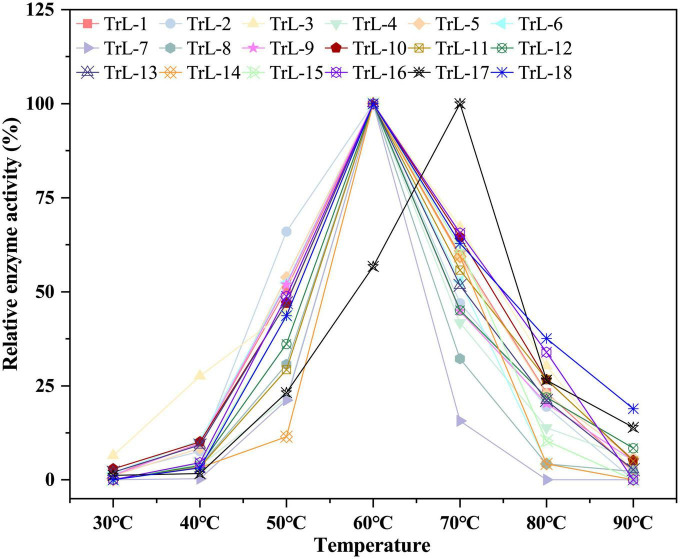
The optimal temperature of chimeras. The chimeras had a low optimal temperature of 70°C for TrL17 and 60°C for the other chimeras, which was lower than that of TrLipE with an optimal temperature of 85°C.

Figure 1 illustrates that the chimeras had a low optimal temperature of 70°C for TrL17 and 60°C for the other chimeras, which was lower than that of TrLipE with an optimal temperature of 85°C (Kay et al., 2016). The half-life of 18 chimeras was in the range of 5–9 h at the optimal temperature, which was also lower than that of TrLipE (>12 h) shown in Supplementary Table 4. Although the half-lives of chimeras increased when the temperature was 10°C lower than the optimal temperature, the same phenomenon of slightly lower thermostability of the chimeras relative to the wild-type TrLipE was observed. The common feature shared by chimeras and TrLipE was that they nearly completely lost their enzymatic activity after 3 h when the temperature was 10°C higher than the optimal temperature. Therefore, the above results indicate that the lid affects the thermostability of the chimeras, which has also been confirmed by other studies. Yu et al. (2014) found that lid swapping has a negative effect on the thermostability of chimeras when studying the conversion of lipase to esterase. Coincidentally, Huang et al. (2019) indicated an obvious decrease in Tm and half-life values of chimera (AFLB-CALBlid) obtained by lid swapping. As reported by Khan et al. (2017), the thermostability of lipases can be altered by modifications in their lid domains because thermophilic lipases generally have a large and rigid lid structure. Nevertheless, these chimeras are less thermostable than TrLipE and superior to other reported lipases (Yang et al., 2015, 2016; Li et al., 2022).

**FIGURE 2:**
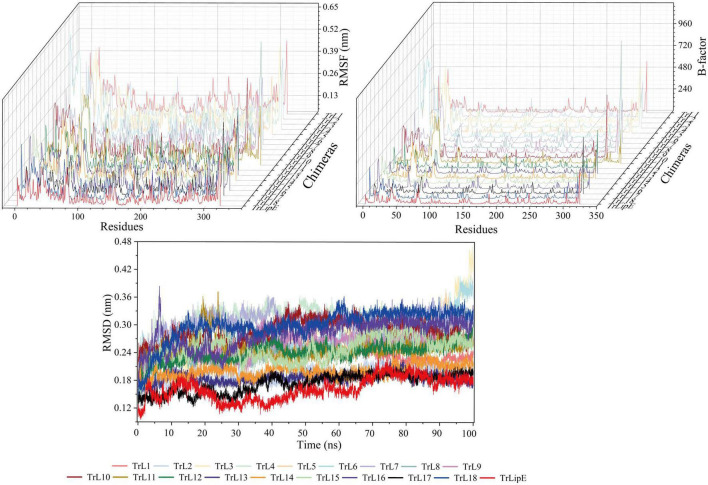
The MD simulation result of TrLipE and chimeras. MD simulations were performed at 300 K, and the chimeras had higher RMSD, RMSF, and B-factor values than TrLipE.

To further evaluate the effect of the lid area on thermostability, MD simulations were performed at 300 K. The Root Mean Square Deviation (RMSD), Root Mean Square Fluctuation (RMSF), and B-factor values were analyzed, which represent the flexibility of the protein structure. Higher values indicate greater flexibility of the protein structure and vice versa, so they can characterize the thermostability of the protein. As shown in Figure 2, the results clearly demonstrated that chimeras had higher RMSD, RMSF, and B-factor values than TrLipE, which indicated that chimeras had greater flexibility and poorer thermostability than wild-type TrLipE. However, although the chimeras had higher average RMSD (0.174 ± 0.052 nm), RMSF (0.126 ± 0.037 nm), and B-factor (53.13 ± 3.18) values than TrLipE, the values for TrL17 were lower than those of other chimeras (Supplementary Figure 1), thus confirming the optimal temperature results mentioned above.

**FIGURE 3:**
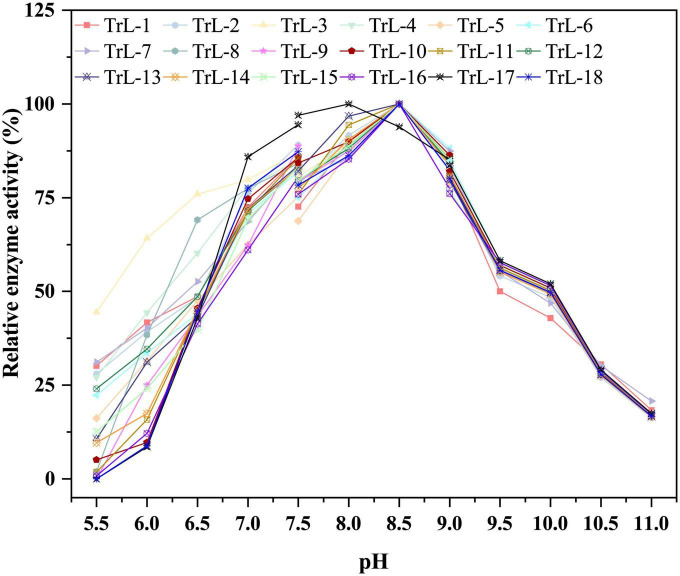
The optimal pH of chimeras. The optimal pH for the chimeras was 8–8.5, and this value was slightly lower than the pH of 8.5–9 for TrLipE.

Compared to the thermostability of chimeras, the optimum pH did not change significantly. Figure 3 illustrates the optimal pH of the chimeras and TrLipE. The results showed that lid swapping had a weak effect on the pH of the chimeras. The optimal pH for the chimeras was 8–8.5, and this value was slightly lower than the pH of 8.5–9 for TrLipE, which was similar to that of most alkaline lipases (Melani et al., 2019).

**FIGURE 4:**
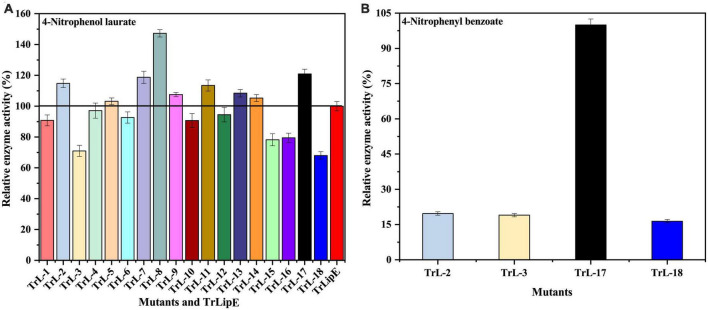
The relative enzyme activity of TrLipE and chimeras. **(A)** The relative enzyme activity of TrLipE and chimeras for 4-nitrophenol laurate; **(B)** The relative enzyme activity of chimeras TrL2, TrL3, TrL,17, and TrL18 for 4-nitrophenyl benzoate.

To test the enzymatic activity, 4-nitrophenol laurate was used as a substrate. Figure 4A shows the specific enzymatic activities of the chimeras. All chimeras were able to hydrolyze the substrate 4-nitrobenzene laurate, but their activity changed. Chimeras TrL2, TrL5, TrL7, TrL8, TrL9, TrL11, TrL13, TrL14, and TrL17 were increased, with TrL17 and TrL8 representing the best performing chimeras and showing an increase in specific enzyme activity by approximately 20 ± 3.043% and 47 ± 2.361%, respectively. However, the other chimeras showed an opposite trend compared to TrLipE, which might be caused by the different lid structure on the binding ability of substrate 4-nitrophenol laurate. In addition, regarding the substrate specificity of chimeras, TrL2, TrL3, TrL17, and TrL18 could specifically recognize and hydrolyze 4-nitrophenyl benzoate, whereas TrLipE and the other chimeras could not. TrL17 had a specific enzyme activity of 0.5 ± 0.0247 μmol⋅mg^–1^⋅min^–1^ p-nitrophenol when 4-nitrophenyl benzoate was used as the substrate at a concentration of 150 μmol⋅L^–1^, and this activity was higher than that of TrL2, TrL3, and TrL18, as shown in Figure 4B, the specific enzymatic activity of chimera TrL2, TrL3 and TrL18 was only 19.68 ± 0.72%, 18.98 ± 0.70% and 16.41 ± 0.68% of TrL17, respectively. Then, we also used 4-nitrophenyl benzoate analogies (4-nitrophenyl anthranilate and 4-nitrophenyl salicylate) as substrates and found that all chimeras did not hydrolyze these two substrates, which was similar to the findings for TrLipE. As reported by Brocca et al. (2003), it could be concluded that the role of the lid in affecting the enzyme activity and specificity of chimeras might be complex, which has also been demonstrated previously (Akoh et al., 2004; Skjøt et al., 2009; Vagstad et al., 2012).

### 3.3. Determination of the kinetic parameters of the chimeras and TrLipE

To analyze the changes in the affinity of the chimeras for different substrates, p-NP acetate (C2), p-NP butyrate (C4), p-NP hexanoate (C6), p-NP caprylate (C8), p-NP decanoate (C10), p-NP laurate (C12), p-NP palmitate (C16), and p-NP benzoate were used to determine the kinetic parameters of the chimeras and TrLipE. The values of *K*_*m*_, *k*_*cat*_, and *k*_*cat*_/*K*_*m*_ for the chimeras and TrLipE are listed in Tables 2–4, respectively, and the fitted curves are shown in Supplementary Figure 2. The results indicated that the 18 chimeras had different affinities and catalytic abilities for different substrates and showed characteristics similar to those of TrLipE; that is, they showed better affinity for substrates with longer carbon chains. Among the tested substrates, 15 chimeras (except TrL7, TrL13, and TrL16) showed decreased activity for 4-nitrophenol palmitate and had a minimum *K*_*m*_ value in the range of 7.46–77.35 μmol⋅L^–1^, which was lower than the 553.19–861.37 μmol⋅L^–1^ for 4-nitrophenol acetate. However, despite the good affinity for 4-nitrophenol palmitate, the chimeras TrL1, TrL5, TrL6, TrL8, TrL10, and TrL11 had lower kcat and *k*_*cat*_/*K*_*m*_ values (Tables 3, 4) compared to that obtained for the other substrates and chimeras. This may be because high affinity slows the release of the product after lipase hydrolysis. Regarding the other substrates, the chimeras exhibited obvious differences in *K*_*m*_, *k*_*cat*_, and *k*_*cat*_/*K*_*m*_ values, suggesting that the lid played an important role in the specificity or selectivity of the lipase (Brocca et al., 2003).

**TABLE 2:** The *K*_*m*_ value of chimeras and TrLipE.

Mutants	4-Nitrophenyl acetate	4-Nitrophenyl butyrate	4-Nitrophenyl hexanoate	4-Nitrophenyl caprylate	4-Nitrophenyl decanoate	4-Nitrophenyl laurate	4-Nitrophenyl palmitate	4-Nitrophenyl benzoate
	*K*_*m*_ (μmol/L)							
TrL1	845.20	662.20	1135.04	187.31	69.73	63.17	55.30	
TrL2	632.80	863.92	748.86	644.78	219.97	107.94	24.53	50.03
TrL3	633.79	829.22	797.08	769.30	184.4	280.76	36.67	36.99
TrL4	785.43	723.48	1060.08	189.67	77.49	55.64	7.46	
TrL5	609.85	306.11	282.59	79.57	40.44	21.12	31.49	
TrL6	567.92	457.48	748.29	136.71	55.41	46.20	45.91	
TrL7	861.37	93.63	315.28	64.05	14.21	12.76		
TrL8	558.10	315.89	206.90	56.16	21.84	30.56	46.87	
TrL9	553.19	221.50	309.40	73.85	61.91	43.35	23.15	
TrL10	561.39	716.72	781.68	279.01	113.24	62.28	66.50	
TrL11	572.57	886.03	703.93	374.62	168.37	87.46	77.35	
TrL12	587.96	362.00	366.12	165.43	42.09	44.84	13.72	
TrL13	601.78	626.08	798.13	106.18	77.17	58.89		
TrL14	836.41	664.08	794.05	175.14	72.76	38.60	10.17	
TrL15	620.01	717.16	929.68	325.07	120.76	68.68	12.65	
TrL16	618.58	384.29	489.57	132.71	44.71	64.94		
TrL17	599.21	963.92	928.33	702.94	1701.05	384.43	42.44	60.24
TrL18	653.93	993.28	1074.06	641.11	1014.23	210.49	32.46	44.86
TrLipE	949	8,249	881	593	89.38	57	45	

**TABLE 3:** The *k*_*cat*_ value of chimeras and TrLipE.

Mutants	4-Nitrophenyl acetate	4-Nitrophenyl butyrate	4-Nitrophenyl hexanoate	4-Nitrophenyl caprylate	4-Nitrophenyl decanoate	4-Nitrophenyl laurate	4-Nitrophenyl palmitate	4-Nitrophenyl benzoate
	*k*_*cat*_ (min^–1^)							
TrL1	682.14	421.11	665.46	162.80	98.02	75.46	21.07	
TrL2	590.91	689.09	685.91	560.45	220.91	144.59	34.27	7.7
TrL3	409.38	494.72	506.06	456.00	199.02	203.90	32.56	8.18
TrL4	560.87	379.34	617.96	157.80	91.61	69.7	12.71	
TrL5	585.01	231.38	296.08	108.94	59.04	42.11	6.85	
TrL6	491.00	315.85	560.52	138.22	74.43	49.53	6.93	
TrL7	1357.69	131.18	195.99	75.14	41.79	38.61		
TrL8	779.44	244.57	265.22	102.20	56.73	54.46	13.38	
TrL9	568.22	193.85	303.80	103.13	80.62	54.01	11.94	
TrL10	480.26	510.23	610.25	239.08	116.80	86.48	18.05	
TrL11	610.68	748.32	645.09	363.64	189.84	132.90	24.51	
TrL12	520.48	273.99	317.89	156.97	64.32	48.22	10.20	
TrL13	608.30	456.44	661.66	138.62	104.04	61.31		
TrL14	759.59	443.55	583.49	182.84	113.04	113.04	20.16	
TrL15	454.59	479.83	625.99	248.85	110.02	78.20	13.08	
TrL16	463.02	236.86	363.60	476.30	57.43	61.95		
TrL17	701.18	597.74	588.88	456.65	933.41	290.08	35.88	21.92
TrL18	413.33	555.45	607.15	376.80	524.79	164.68	26.57	7.89
TrLipE	704.65	645.82	290.93	239.86	88.38	125.38	33.76	

**TABLE 4:** The *k_*cat*_/K_*m*_* value of chimeras and TrLipE.

Mutants	4-Nitrophenyl acetate	4-Nitrophenyl butyrate	4-Nitrophenyl hexanoate	4-Nitrophenyl caprylate	4-Nitrophenyl decanoate	4-Nitrophenyl laurate	4-Nitrophenyl palmitate	4-Nitrophenyl benzoate
	*k_*cat*_/K_*m*_* (L⋅min^–1^⋅mmol^–1^)							
TrL1	807.08	635.93	586.29	869.15	1405.71	1194.55	381.01	
TrL2	933.80	797.63	915.94	869.21	1004.27	1339.54	1397.07	153.91
TrL3	645.92	596.61	634.89	592.75	1079.28	726.24	887.92	221.14
TrL4	714.09	524.33	582.94	831.97	1182.22	1252.70	1703.75	
TrL5	959.27	755.87	1047.74	1369.11	1459.94	1993.85	217.53	
TrL6	864.56	690.41	749.07	1011.05	1343.26	1072.08	150.95	
TrL7	1576.20	1401.05	621.64	1173.15	2940.89	3025.86		
TrL8	1396.60	774.23	1281.88	1819.80	2597.53	1782.07	285.47	
TrL9	1027.17	875.17	981.90	1396.48	1302.21	1245.91	515.77	
TrL10	855.48	711.90	780.69	856.89	1031.44	1388.57	271.43	
TrL11	1066.56	844.58	916.41	970.69	1127.52	1519.55	316.87	
TrL12	885.23	756.88	868.27	948.86	1528.15	1075.38	743.44	
TrL13	1010.84	729.04	829.01	1305.52	1348.19	1041.09		
TrL14	908.16	667.92	734.83	1043.97	1553.60	2928.50	1982.30	
TrL15	733.20	669.07	673.34	765.53	911.06	1138.61	1033.99	
TrL16	748.52	616.36	742.69	3589.03	1284.50	953.96		
TrL17	1170.17	620.11	634.34	649.63	548.73	754.57	845.43	363.88
TrL18	632.07	559.21	565.29	587.73	517.43	782.37	818.55	175.88
TrLipE	742.52	78.29	330.23	404.49	988.81	1444.62	745.02	

An interesting result observed for most of the chimeras and TrLipE was the affinity for p-nitrophenyl butyrate (C4). Although the chimeras had a lower *K*_*m*_ value and higher *k*_*cat*_ and *k*_*cat*_/*K*_*m*_ values (Tables 3, 4) for 4-nitrophenol butyrate compared to TrLipE, most chimeras still possessed a poor affinity for this substrate compared to that for 4-nitrophenyl acetate. However, some chimeras (TrL5, TrL6, TrL7, TrL8, TrL9, TrL12, and TrL16) exhibited better affinity for 4-nitrophenol butyrate. This further confirmed that the effect of the lid on lipase specificity is complex.

With respect to substrate specificity, chimeras TrL2, TrL3, TrL17, and TrL18 could specifically hydrolyze 4-nitrophenyl benzoate while TrLipE and other chimeras could not. Although TrL17 had a higher *K*_*m*_ value of 60.24 μmol⋅L^–1^ compared to the other three chimeras (50.03, 36.99, and 44.86 μmol⋅L^–1^, respectively), TrL17 exhibited higher activity and catalytic capability, with *k*_*cat*_ and *k*_*cat*_/*K*_*m*_ values of 21.92 min^–1^ and 363.88 L⋅min^–1^⋅mmol^–1^, respectively, which were higher than those of the chimeras TrL2, TrL3, and TrL18. As confirmed by the results of the kinetic study, the lid is involved in the substrate specificity of lipase, which has also been demonstrated by other studies (Vanleeuw et al., 2019).

### 3.4. Docking and site-directed mutagenesis of TrL17

The binding pocket is an important target for modifying the catalytic activity of enzymes (Ma et al., 2022). Thus, to improve the accessibility and catalytic efficiency of TrL17 for 4-nitrophenyl benzoate, molecular docking simulations were performed using Schrödinger software, which is a powerful simulation tool for evaluating protein–ligand interactions (Anuar et al., 2021). From Supplementary Figure 3, the serine of the active center acts as a nucleophile on the ester bond, as previously reported (Cen et al., 2019), and G84 and G85 participate in the formation of oxygen holes. Furthermore, other interactions, such as C-H bonds, pi-alkyl, and Pi-Pi bonds, were formed between ligands and other amino acids involved in docking. Therefore, based on the results of docking, the “Build and Edit Protein” protocol of DS was applied to perform the mutation and binding energy calculations. Then, 72 single mutations were selected based on the lower binding energy.

Based on the above method of kinetic parameters determination, enzymatic reactions were determined in accordance with assays conditions described above and carried out with 4-nitrophenyl benzoate as the substrate, and the *K*_*m*_, *k*_*cat*_, and catalytic efficiency (*k*_*cat*_/*K*_*m*_) values of the 72 single mutants are summarized in Supplementary Table 5. Most of the mutants reduced the catalytic activity of 4-nitrophenyl benzoate, for example, the catalytic efficiency of the mutant G86L (15.509 L⋅mmol^–1^⋅min^–1^) was reduced by 20-fold compared to the wild-type TrL17 (331.39 L⋅mmol^–1^⋅min^–1^), and 22 mutants lost their activity directly. In these mutants, such as A158, A156, A254, L256, F257, F284, and G286, residues were mainly distributed around catalytic triplets (S157, D255, and H285). In addition, the mutants of G84 and G85, which are involved in the formation of catalytic tetrahedral structures, also lost their activity. These results suggest that residues in the conserved region play a critical role in enzyme activity (Nezhad et al., 2023).

**FIGURE 5:**
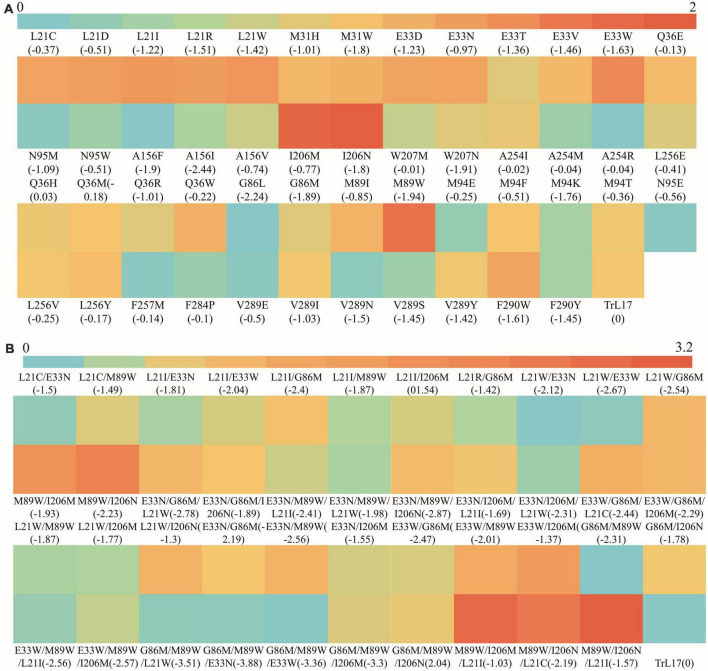
The heat map of the ratio of *k*_*cat*_/*Km* value between TrL17 and mutants. **(A)** The ratio of *k*_*cat*_/*Km* values between single mutants and TrL17; **(B)** The ratio of *k*_*cat*_/*Km* values between double, triple mutants, and TrL17.

The heat map shown in Figure 5A demonstrates the ratio of *k*_*cat*_/*K*_*m*_ values between single mutants (except for those showing a loss of enzyme activity) and TrL17. The figure clearly shows that the *k*_*cat*_/*K*_*m*_ value of 10 mutants at six residue sites were 1.28- to 2.23-fold higher than that of the wild-type TrL17, with mutant M89W (2.23-fold higher) having a highest *k*_*cat*_/*K*_*m*_ value of 601.42 L⋅mmol^–1^⋅min^–1^ and showing the greatest improvement compared to the other mutations. Compared with that of TrL17, the tryptophan of mutant M89W formed a Pi-Pi interaction with the substrate and the binding energy changed from 0 to −1.94 kcal⋅mol^–1^, which led to an increase in binding affinity and thus an increase in enzyme activity. The binding energy plays a pivotal role in *K*_*m*_ and *k*_*cat*_; therefore, reducing the binding energy of the protein and ligand complex has a positive effect on improving the efficiency of enzyme catalysis (Dutta Banik et al., 2016; Wang et al., 2020). In our study, we also found that reducing the binding energy could improve enzyme activity, although this does not mean that the binding energy could be reduced indefinitely. The results showed that the catalytic efficiency of the mutant increased and then decreased with a decrease in the binding energy when the mutation was carried out at the same site (such as L21). The reason for this phenomenon may be that after the enzyme reaction, the product could not be released quickly owing to the low binding energy. In addition, we did not identify a specific relationship between the binding energy and catalytic efficiency of the mutants from different sites.

To further improve the catalytic efficiency of TrL17 on 4-nitrophenyl benzoate, double and triple substitution variants were designed according to the results of the single substitution. After enzymatic reaction, the *K*_*m*_, *k*_*cat*_, and catalytic efficiency (*k*_*cat*_/*K*_*m*_) values of the 43 mutants (double and triple substitutions) are calculated and shown in Supplementary Table 6. Figure 5B displays the heat map of the ratio of *k*_*cat*_/*K*_*m*_ values between the double and triple mutants with TrL17. The results clearly show that increases in substitution sites further reduced the binding ability. For example, the double mutation L21W/E33W (365.98 L⋅mmol^–1^⋅min^–1^) and triple mutation E33N/G86M/M89W (365.64 L⋅mmol^–1^⋅min^–1^) had the lowest binding energies of −2.67 kcal⋅mol^–1^ and −3.88 kcal⋅mol^–1^, respectively; moreover, their catalytic performance on 4-nitrophenyl benzoate was not the best. Among the double mutants, M89W/I206M (833.58 L⋅mmol^–1^⋅min^–1^) and M89W/I206N (921.8 L⋅mmol^–1^⋅min^–1^) showed excellent performance, and their catalytic efficiencies were 2.51- and 2.78-fold higher than those of TrL17, respectively, and showed corresponding increases compared to those of the single mutation. Among the triple mutations, M89W/I206M/L21I (1019.93 L⋅mmol^–1^⋅min^–1^) and M89W/I206N/L21I (1058.89 L⋅mmol^–1^⋅min^–1^) were the best, and their catalytic efficiencies were up to 3.08- and 3.20-fold higher than those of TrL17, respectively. In addition, the catalytic efficiency of the other double and triple mutants was also improved compared to that of TrL17. Our study shows that it is feasible to improve the catalytic efficiency of enzymes by reducing the binding energy of protein–ligand complexes.

## 4. Conclusion

In this study, 18 chimeras were successfully constructed by lid swapping. Most of the chimeras showed higher expression levels than the wild TrLipE. Meanwhile, the MD simulation results showed that the chimeras had greater flexibility and relatively lower optimal temperatures, such as 70°C for TrL17 and 60°C for the other chimeras, compared with TrLipE, and they also had a poorer half-life than TrL17. Moreover, compared to other reported thermostable lipases, the thermostability of these 18 chimeras was excellent. In addition, they could still withstand a wide pH range, which indicates that they still have great potential for future applications in extreme conditions, such as the detergent industry and biodiesel industry. Among the tested substrates, the specific enzyme activity and affinity of the partial chimeras were higher than those of the wild TrLipE, due to the change of substrate binding ability of chimeras after lid swapping. The chimeras TrL2, TrL3, TrL17, and TrL18 had better characteristics, and they could specifically hydrolyze 4-nitrophenyl benzoate, which cannot be hydrolyzed by TrLipE, with TrL17 showing the best ability to hydrolyze 4-nitrophenyl benzoate. After changing the substrate specificity of the chimera by lid swapping, we further optimized the catalytic center of the chimera by relying on the strategy of reducing the enzyme-substrate binding energy, and finally the catalytic efficiency of chimera TrL17 mutation was up to 3.20-fold faster than that of wild TrL17, which provided certain support for the further application of the chimera TrL17, and also provided valuable insights into the rational design of other lipases.

## Data availability statement

The original contributions presented in this study are included in the article/Supplementary material, further inquiries can be directed to the corresponding authors.

## Author contributions

YF: investigation, formal analysis, and writing—original draft. FL and TY: methodology, validation, and investigation. YS: writing—review and editing. YX: methodology and software. ZG: data curation and visualization. GS: supervision and methodology. LZ: funding acquisition, project administration, and methodology. All authors contributed to the article and approved the submitted version.
