# Supplementary material for: N-terminal lid swapping contributes to the substrate specificity and activity of thermophilic lipase TrLipE
Source: Front Microbiol. 2023 Jun 26;14:1193955. doi: 10.3389/fmicb.2023.1193955 (PMC10332459; doi:10.3389/fmicb.2023.1193955)
Supplement: Supplementary file 1 [file Data_Sheet_1.pdf]

Supplementary Material

N-terminal Lid Swapping Contributes to the Substrate Specificity and Activity of Thermophilic Lipase TrLipE

Yakun Fang [1,2], Fan Liu [1,2], Yi Shi [1,2], Ting Yang [3], Yu Xin[1,2,*], Zhenghua Gu [1,2], Guiyang Shi [1,2], Liang Zhang [1,2,*]

*** Correspondence:** Liang Zhang, email address: zhangl@jiangnan.edu.cn, telephone/fax number: +86 051085918235;

Yu Xin, email address: yuxin@jiangnan.edu.cn, telephone/fax number: +86 051085918119;

1 Supplementary Figures and Tables

1.1 Supplementary Figures

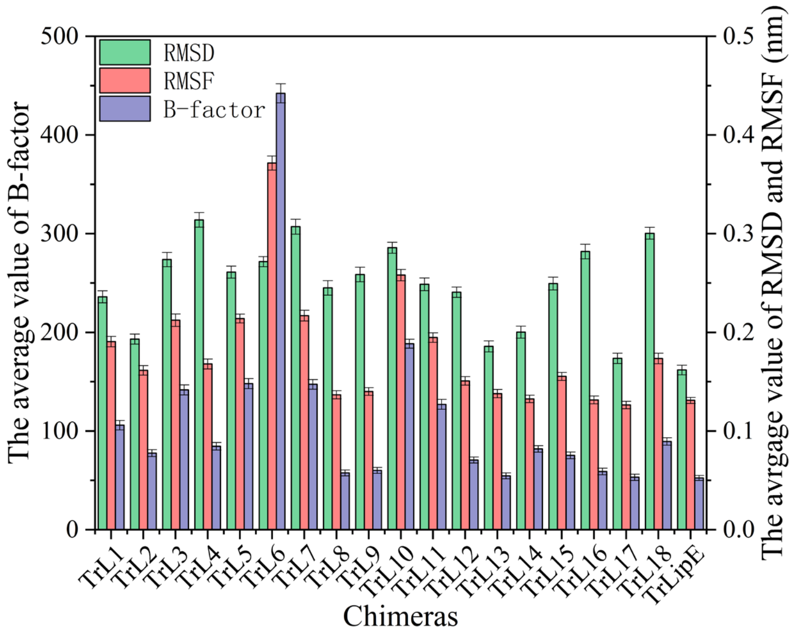

Supplementary Figure 1. The RMSD, RMSF and B-factor average value of TrLipE and chimeras

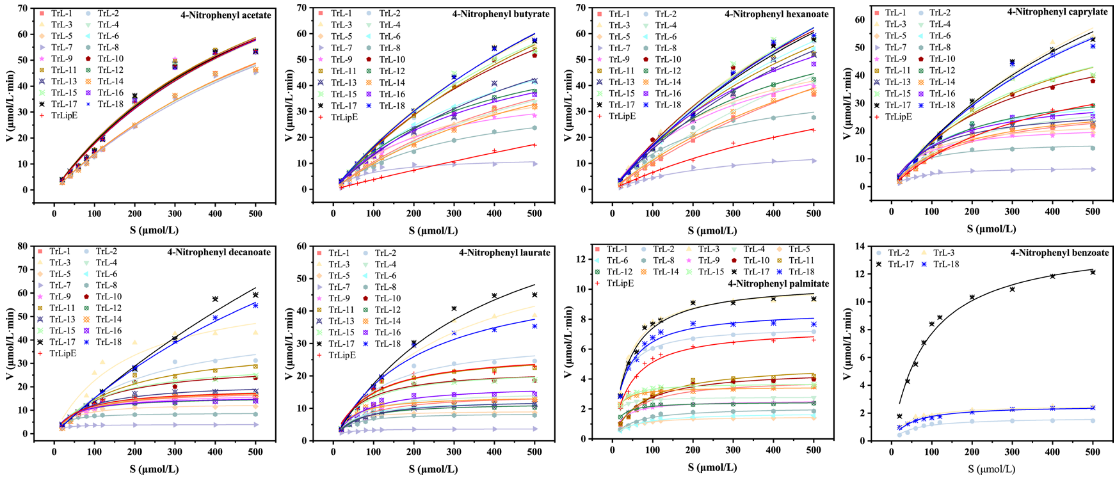

Supplementary Figure 2 Fitting curve of substrate concentration and initial velocity

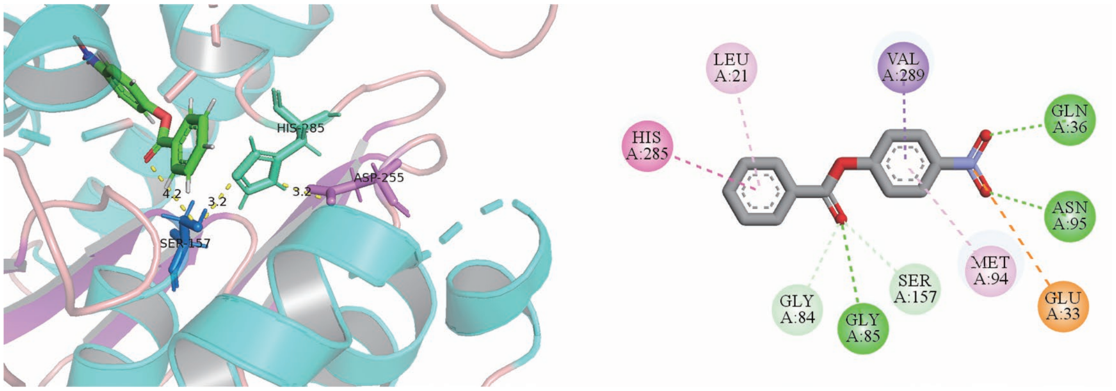

Supplementary Figure 3 The molecule docking between TrL17 and 4-nitrophenyl benzoate

1.2 Supplementary Table

Supplementary Table 1 The primers sequence for chimeras

Primers	5’-3’		
R-1	TTAGAATTCCTCCTTACCGGATCCCGC	R-2	CAACTTGGCGTTGATCGTGAACG
1VEQ-F	GGTAAGGAGGAATTCTAAATGCTGGATCCAGTGATTCAAC	1VEQ-R	TCACGATCAACGCCAAGTTGCTCGGCTCTCTCGGCCACCC
3HIB-F	CCGGTAAGGAGGAATTCTAAATGGCCGGGCCCGAGATCGT	3HIB-R	TCACGATCAACGCCAAGTTGCTCGGCTCTCTCGGCCACCC
3K6K-F	AAGGAGGAATTCTAAATGGATACAAAAATGGATCCTAGAG	3K6K-R	TCACGATCAACGCCAAGTTGTTCGGCTCTCTCGGCCACCC
3QH4-F	CCGGTAAGGAGGAATTCTAAATGGTGACACAGCCTGAAGC	3QH4-R	CGATCAACGCCAAGTTGTTCAGCTCTTTCAGCCACCCATC
4C89-F	TAAGGAGGAATTCTAAATGAATGATATTGCCGGAGAACAG	4C89-R	TCACGATCAACGCCAAGTTGCTCGGCTCTCTCGGCCACCC
4N5H-F	GGTAAGGAGGAATTCTAAATGGCTGATGAAGAAGCCATGC	4N5H-R	CGATCAACGCCAAGTTGTTCAGCTCTTTCAGCCACCCATC
4P9N-F	TAAGGAGGAATTCTAAATGCCTCTGGATCCTAGAATTAAG	4P9N-R	TCACGATCAACGCCAAGTTGCTCGGCTCTCTCGGCCACCC
4V2I-F	CCGGTAAGGAGGAATTCTAAATGCCCGTGCTGGAGCCCAC	4V2I-R	CGATCAACGCCAAGTTGTTCAGCTCTTTCGGCTACCCATC
4YPV-F	CCGGTAAGGAGGAATTCTAAATGGCCCTGGATCCTCAGGC	4YPV-R	TCACGATCAACGCCAAGTTGCTCGGCTCTCTCGGCCACCC
4ZRS-F	GGTAAGGAGGAATTCTAAATGACCCCCGAGCTGAGAGC	4ZRS-R	CGATCAACGCCAAGTTGTTCGGCTCTTTCAGCCACCCATC
5HC0-F	GTAAGGAGGAATTCTAAATGAAGATCGCCGAGGACCC	5HC0-R	TCACGATCAACGCCAAGTTGCTCAGCTCTTTCAGCCACCC
5MIF-F	CCGGTAAGGAGGAATTCTAAATGCAGCTGGACCCCATCAC	5MIF-R	TCACGATCAACGCCAAGTTGCTCGGCTCTCTCAGCCACCC
6AAE-F	GGTAAGGAGGAATTCTAAATGCCTCTGAATCCTCATGTGG	6AAE-R	TCACGATCAACGCCAAGTTGCTCGGCCCGCTCGGCCACCC
6K34-F	GGTAAGGAGGAATTCTAAATGAGACCCGCCCTGGACGC	6K34-R	TCACGATCAACGCCAAGTTGTTCGGCTCTTTCAGCCACCC
6RJ8-F	GTAAGGAGGAATTCTAAATGGACGAGATCGTGTTCGACC	6RJ8-R	TCACGATCAACGCCAAGTTGTTCGGCTCTCTCGGCCACCC
7B1X-F	GTAAGGAGGAATTCTAAATGAACGGCAACGACCCCGAC	7B1X-R	TCACGATCAACGCCAAGTTGCTCGGCTCTCTCAGCTACCC
7B4Q-F	GGTAAGGAGGAATTCTAAAGAAGCTTGCGGCCGCACTCGA	7B4Q-R	TCACGATCAACGCCAAGTTCCTCCTTACCGGATCCCGCC
7UAY-F	GGTAAGGAGGAATTCTAAATGGCCTCTGAAGCTCTGACA	7UAY-R	TCACGATCAACGCCAAGTTGTTCAGCTCTTTCGGCCACCC

Supplementary Table 2 The primers sequence for mutants

Primers	5'-3'		
L21C-F	CCGCCGCTTAACTGTGACGATGTGCAGGCCACTCG	L21C-R	GGCCTGCACATCGTCACAGTTAAGCGGCGGAAACA
L21D-F	CCGCCGCTTAACGATGACGATGTGCAGGCCACTCG	L21D-R	GGCCTGCACATCGTCATCGTTAAGCGGCGGAAACA
L21I-F	CCGCCGCTTAACATCGACGATGTGCAGGCCACTCG	L21I-R	CCTGCACATCGTCGATGTTAAGCGGCGGAAACATA
L21R-F	CCGCCGCTTAACCGTGACGATGTGCAGGCCACTCG	L21R-R	GGCCTGCACATCGTCAGCGTTAAGCGGCGGAAACA
L21W-F	CCGCCGCTTAACTGGGACGATGTGCAGGCCACTCG	L21W-R	CCTGCACATCGTCCCAGTTAAGCGGCGGAAACATA
M31H-F	CCACTCGTAAGGCGCACGAAGAAGCGGCGCAATTG	M31H-R	GCCGCTTCTTCGTGCGCCTTACGAGTGGCCTGCAC
M31W-F	CCACTCGTAAGGCGTGGGAAGAAGCGGCGCAATTG	M31W-R	GCCGCTTCTTCCCACGCCTTACGAGTGGCCTGCAC
E33D-F	CGTAAGGCGATGGAAGATGCGGCGCAATTGACGG	E33D-R	CAATTGCGCCGCATCTTCCATCGCCTTACGAGTGG
E33T-F	CGTAAGGCGATGGAAACGGCGGCGCAATTGACGG	E33T-R	CAATTGCGCCGCCGTTTCCATCGCCTTACGAGTGG
E33N-F	CGTAAGGCGATGGAAAATGCGGCGCAATTGACGG	E33N-R	CAATTGCGCCGCATTTTCCATCGCCTTACGAGTGG
E33V-F	CGTAAGGCGATGGAAGTGGCGGCGCAATTGACGG	E33V-R	CAATTGCGCCGCCACTTCCATCGCCTTACGAGTGG
E33W-F	GTAAGGCGATGGAATGGGCGGCGCAATTGACGGA	E33W-R	CAATTGCGCCGCCCATTCCATCGCCTTACGAGTGG
Q36E-F	GGAAGAAGCGGCGGAATTGACGGAGTTGCCGGTG	Q36E-R	CGGCAACTCCGTCAATTCCGCCGCTTCTTCCATCG
Q36H-F	GAAGCGGCGCACTTGACGGAGTTGCCGGTGGAT	Q36H-R	GGCAACTCCGTCAAGTGCGCCGCTTCTTCCATCG
Q36M-F	GGAAGAAGCGGCGATGTTGACGGAGTTGCCGGTG	Q36M-R	GGCAACTCCGTCAACATCGCCGCTTCTTCCATCG
Q36R-F	GGAAGAAGCGGCGCGTTTGACGGAGTTGCCGGTG	Q36R-R	GGCAACTCCGTCAAACGCGCCGCTTCTTCCATCG
Q36W-F	GGAAGAAGCGGCGTGGTTGACGGAGTTGCCGGTG	Q36W-R	GGCAACTCCGTCAACCACGCCGCTTCTTCCATCG
G84D-F	GTATTATTGTGGATTCACGATGGTGGGTATGTTAT	G84D-R	CATAACATACCCACCATCGTGAATCCACAATAAT
G84T-F	CAGTATTATTGTGGATTCACACGGGTGGGTATGTT	G84T-R	CATAACATACCCACCCGTGTGAATCCACAATAATA
G84R-F	GTATTATTGTGGATTCACCGTGGTGGGTATGTTAT	G84R-R	CCATAACATACCCACCACGGTGAATCCACAATAA
G84V-F	CCAGTATTATTGTGGATTCACGTGGGTGGGTATGT	G84V-R	ATAACATACCCACCCACGTGAATCCACAATAATAC
G85D-F	GTGGATTCACGGAGATGGGTATGTTATGGGCGCTC	G85D-R	CATAACATACCCATCTCCGTGAATCCACAATAATA
G85F-F	GTGGATTCACGGATTTGGGTATGTTATGGGCGCTC	G85F-R	CATAACATACCCAAATCCGTGAATCCACAATAATA
G85N-F	GTGGATTCACGGAAATGGGTATGTTATGGGCGCTC	G85N-R	CATAACATACCCATTTCCGTGAATCCACAATAATA
G85S-F	GTGGATTCACGGATCGGGGTATGTTATGGGCGCTC	G85S-R	CATAACATACCCCGATCCGTGAATCCACAATAATA
G85V-F	GTGGATTCACGGAGTGGGGTATGTTATGGGCGCTC	G85V-R	CATAACATACCCCACTCCGTGAATCCACAATAATA
G86L-F	GTGGATTCACGGAGGTCTTTATGTTATGGGCGCTC	G86L-R	AGCGCCCATAACATAAAGACCTCCGTGAATCCACA
G86M-F	TGGATTCACGGAGGTATGTATGTTATGGGCGCTC	G86M-R	GAGCGCCCATAACATACATACCTCCGTGAATCCAC
G86Q-F	TGGATTCACGGAGGTCAATATGTTATGGGCGCTC	G86Q-R	AGCGCCCATAACATATTGACCTCCGTGAATCCACA
M89I-F	GAGGTGGGTATGTTATCGGCGCTCCGGAAATGAA	M89I-R	CCGGAGCGCCGATAACATACCCACCTCCGTGAATCC
M89W-F	GAGGTGGGTATGTTTGGGGCGCTCCGGAAATGAA	M89W-R	CCGGAGCGCCCCAAACATACCCACCTCCGTGAATCC
M94E-F	GGGCGCTCCGGAAGAAAATGATCAGCAATGTGCA	M94E-R	ATTGCTGATCATTTTCTTCCGGAGCGCCCATAACAT
M94F-F	GGGCGCTCCGGAATTTAATGATCAGCAATGTGCA	M94F-R	ATTGCTGATCATTAAATTCCGGAGCGCCCATAACAT
M94K-F	GGGCGCTCCGGAAAAAAATGATCAGCAATGTGCA	M94K-R	ATTGCTGATCATTAATTTCCGGAGCGCCCATAACAT
M94L-F	GGGCGCTCCGGAACTTAATGATCAGCAATGTGCA	M94L-R	ATTGCTGATCATTAAGTTCCGGAGCGCCCATAACAT
M94T-F	GGGCGCTCCGGAAACGAATGATCAGCAATGTGCA	M94T-R	ATTGCTGATCATTCGTTTCCGGAGCGCCCATAACAT
N95E-F	CGCTCCGGAAATGGAAGATCAGCAATGTGCAGAG	N95E-R	GCACATTGCTGATCTTCCATTTCCGGAGCGCCCATA
N95M-F	CGCTCCGGAAATGATGGATCAGCAATGTGCAGAG	N95M-R	GCACATTGCTGATCCATCATTTCCGGAGCGCCCATA
N95R-F	CGCTCCGGAAATGCGTGATCAGCAATGTGCAGAG	N95R-R	GCACATTGCTGATCACGCATTTCCGGAGCGCCCATA
N95T-F	CGCTCCGGAAATGACGGATCAGCAATGTGCAGAG	N95T-R	GCACATTGCTGATCCGTCATTTCCGGAGCGCCCATA
N95W-F	CGCTCCGGAAATGTGGGATCAGCAATGTGCAGAG	N95W-R	GCACATTGCTGATCCCACATTTCCGGAGCGCCCATA
A156F-F	GCAATTGCCGGGTTTTCTGCGGGAGGTGGACTTGC	A156F-R	CCTCCCGCAGAAAACCCGGCAATTGCCAGACGTTC

A156I-F	GCAATTGCCGGGATCTCTGCGGGAGGTGGACTTGC	A156I-R	CCTCCCGCAGAGATCCCGGCAATTGCCAGACGTTC
A156V-F	GCAATTGCCGGGGTGTCTGCGGGAGGTGGACTTG	A156V-R	CCTCCCGCAGACACCCCGGCAATTGCCAGACGTT
A158F-F	GCCGGGGCGTCTTTTGGAGGTGGACTTGCGGCGG	A158F-R	CAAGTCCACCTCCAAAAGACGCCCCGGCAATTGC
A158L-F	GCCGGGGCGTCTCTTGGAGGTGGACTTGCGGCGG	A158L-R	CAAGTCCACCTCCAAGAGACGCCCCGGCAATTGC
A158V-F	GCCGGGGCGTCTGTGGGAGGTGGACTTGCGGCGG	A158V-R	CAAGTCCACCTCCCACAGACGCCCCGGCAATTGC
Y185F-F	CGCTTTCAATTGTTGATCTTTCCGATGTTGGACGA	Y185F-R	CGTCCAACATCGGAAAGATCAACAATTGAAAGC
Y185W-F	CGCTTTCAATTGTTGATCTGGCCGATGTTGGACGA	Y185W-R	CGTCCAACATCGGCCAGATCAACAATTGAAAGC
I206M-F	CCGATCCACGTCTTATGTGGACCCGCGATTGGAAT	I206M-R	CGCGGGTCCACATAAGACGTGGATCGGTGATTTC
I206N-F	CCGATCCACGTCTTCAATGGACCCGCGATTGGAAT	I206N-R	CGCGGGTCCATTGAAGACGTGGATCGGTGATTTC
W207M-F	CACCGATCCACGTCTTATTATGACCCGCGATTGGA	W207M-R	CAGATTCCAATCGCGGGTCATAATAAGACGTGGA
W207N-F	CACCGATCCACGTCTTATTCAAACCCGCGATTGGA	W207N-R	CAGATTCCAATCGCGGGTTTGAATAAGACGTGGA
A254I-F	CCTACGTCTTAGTAGGAACCATCGATCTTTTCCGC	A254I-R	CGTCGCGGAAAAGATCGATGGTTCCTACTAAGAC
A254M-F	CCTACGTCTTAGTAGGAACCATGGATCTTTTCCGC	A254M-R	CGTCGCGGAAAAGATCCATGGTTCCTACTAAGAC
A254R-F	CCTACGTCTTAGTAGGAACCCGTGATCTTTTCCGC	A254R-R	CGTCGCGGAAAAGATCACGGGTTCCTACTAAGAC
L256E-F	GGAACCGCGGATGAATTCCGCGACGAAGACATCG	L256E-R	CTTCGTCGCGGAATTCATCCGCGGTTCCTACTAAG
L256V-F	GGAACCGCGGATGTGTTCCGCGACGAAGACATCG	L256V-R	CTTCGTCGCGGAACACATCCGCGGTTCCTACTAAG
L256Y-F	GGAACCGCGGATTACTTCCGCGACGAAGACATCG	L256Y-R	CTTCGTCGCGGAAGTAATCCGCGGTTCCTACTAAG
F257M-F	GAACCGCGGATCTTATGCGCGACGAAGACATCGC	F257M-R	TCTTCGTCGCGCATAAGATCCGCGGTTCCTACTAA
F257N-F	GAACCGCGGATCTTCAACGCGACGAAGACATCGC	F257N-R	TCTTCGTCGCGTTGAAGATCCGCGGTTCCTACTAA
F284P-F	TATTTGCGGGAGCACCGCATGGGTTTGACGTATTC	F284P-R	AATACGTCAAACCCATGCGGTGCTCCCGCAAATA
F284W-F	TATTTGCGGGAGCATGGCATGGGTTTGACGTATTC	F284W-R	GAATACGTCAAACCCATGCCATGCTCCCGCAAAT
G286D-F	GCGGGAGCATTTCATGATTTTGACGTATTCGCGCC	G286D-R	GGGCGCGAATACGTCAAAATCATGAAATGCTCCC
G286V-F	GCGGGAGCATTTCATGTGTTTGACGTATTCGCGCC	G286V-R	GGGCGCGAATACGTCAAACACATGAAATGCTCCC
V289E-F	CATGGGTTTGACGAATTCGCGCCCACGGCATGGGT	V289E-R	GGGCGCGAATTCGTCAAACCCATGAAATGCTCCC
V289I-F	CATGGGTTTGACATCTTCGCGCCCACGGCATGGGT	V289I-R	GGGCGCGAAGATGTCAAACCCATGAAATGCTCCC
V289N-F	CATGGGTTTGACAATTTCGCGCCCACGGCATGGGT	V289N-R	GGGCGCGAAATTGTCAAACCCATGAAATGCTCCC
V289S-F	CATGGGTTTGACTCGTTCGCGCCCACGGCATGGGT	V289S-R	GGGCGCGAACGAGTCAAACCCATGAAATGCTCCC
V289Y-F	CATGGGTTTGACTACTTCGCGCCCACGGCATGGGT	V289Y-R	GGGCGCGAAGTAGTCAAACCCATGAAATGCTCCC
F290W-F	GGTTTGACGTATGGGCGCCCACGGCATGGGTGTC	F290W-R	GCCGTGGGCGCCCATACGTCAAACCCATGAAATG
F290Y-F	GGTTTGACGTATACGCGCCCACGGCATGGGTGTC	F290Y-R	GCCGTGGGCGCGTATACGTCAAACCCATGAAATG
21W-33N-F	GGCGATGGAAAATGCGGCGCAATTGACGGAGT	21W-33N-R	CAATTGCGCCGCATTTTCCATCGCCTTACGAGTGG
21W-33W-F	GGCGATGGAATGGGCGGCGCAATTGACGGAGT	21W-33W-R	CAATTGCGCCGCCCATTCCATCGCCTTACGAGTGG
21W-86M-F	GGATTCACGGAGGTATGTATGTTATGGGCGCTCC	21W-86M-R	GCGCCCATAACATACATACCTCCGTGAATCCACAA
21W-89W-F	GGTGGGTATGTTTGGGGCGCTCCGGAAATGAATG	21W-89W-R	GGAGCGCCCCAAACATACCCACCTCCGTGAATCCA
21W-206M-F	CCGATCCACGTCTTATGTGGACCCGCGATTGGAA	21W-206M-R	CGCGGGTCCACATAAGACGTGGATCGGTGATTTCA
21W-206N-F	CCGATCCACGTCTTAATTGGACCCGCGATTGGAA	21W-206N-R	CGCGGGTCCAATTAAGACGTGGATCGGTGATTTCA
86M-89W-F	GGTATGTATGTTTGGGGCGCTCCGGAAATGAATG	86M-89W-R	GGAGCGCCCCAAACATACATACCTCCGTGAATCCA
86M-89W-21I-F	CCGCCGCTTAACATCGACGATGTGCAGGCCACTC	86M-89W-21I-R	CTGCACATCGTCGATGTTAAGCGGCGGAAACATAT
86M-89W-21W-F	CCGCCGCTTAACTGGGACGATGTGCAGGCCACTC	86M-89W-21W-R	CTGCACATCGTCCCAGTTAAGCGGCGGAAACATAT
86M-89W-33N-F	GGCGATGGAAAACGCGGCGCAATTGACGGAGTT	86M-89W-33N-R	CAATTGCGCCGCGTTTTCCATCGCCTTACGAGTGG
86M-89W-33W-F	GGCGATGGAATGGGCGGCGCAATTGACGGAGTT	86M-89W-33W-R	CAATTGCGCCGCCCATTCCATCGCCTTACGAGTGG
86M-89W-206M-F	CCACGTCTTATGTGGACCCGCGATTGGAATCTG	86M-89W-206M-R	CGCGGGTCCACATAAGACGTGGATCGGTGATTTC
86M-89W-206N-F	CCACGTCTTAACTGGACCCGCGATTGGAATCTG	86M-89W-206N-R	CGCGGGTCCAGTTAAGACGTGGATCGGTGATTTC
86M-33W-21C-F	CCGCCGCTTAACTGTGACGATGTGCAGGCCACT	86M-33W-21C-R	CTGCACATCGTCACAGTTAAGCGGCGGAAACATAT

Supplementary Table 3 The proportion of hydrophilic amino acids in the Lid region

Chimeras	the proportion of hydrophilic amino acids in the Lid region (%)	Chimeras	the proportion of hydrophilic amino acids in the Lid region (%)
TrL1	52.38	TrL11	45.46
TrL2	42.50	TrL12	46.34
TrL3	53.33	TrL13	31.71
TrL4	50.00	TrL14	42.22
TrL5	43.19	TrL15	50.00
TrL6	54.55	TrL16	47.92
TrL7	43.19	TrL17	50.00
TrL8	39.58	TrL18	43.18
TrL9	35.42	TrLipE	36.54
TrL10	45.95		

Supplementary Table 4 The half-life of 18 chimeras and TrLipE

	50 °C	60 °C	70 °C		50 °C	60 °C	70 °C		50 °C	60 °C	70 °C
TrL1	12 h	6 h	2 h	TrL7	11 h	5 h	2 h	TrL13	15 h	9 h	4 h
TrL2	14 h	7 h	3 h	TrL8	16 h	8 h	3 h	TrL14	14 h	8 h	2 h
TrL3	11 h	5 h	2 h	TrL9	16 h	9 h	4 h	TrL15	15 h	9 h	3 h
TrL4	13 h	6 h	3 h	TrL10	10 h	6 h	<2 h	TrL16	16 h	9 h	5 h
TrL5	11 h	6 h	2 h	TrL11	13 h	7 h	2 h	TrL18	14 h	8 h	4 h
TrL6	8 h	5 h	1 h	TrL12	15 h	9 h	3 h				
	60 °C	70 °C	80 °C						75 °C	85 °C	95 °C
TrL17	>12 h	9 h	3 h					TrLipE	>12 h	>12 h	9 h

Supplementary Table 5 The K_m, k_{cat} and k_{cat}/K_m value of single substitution variants

Mutants	K_m (μmol·L^{-1})	k_{cat} (min^{-1})	k_{cat}/K_m (L·mmol^{-1}·min^{-1})	Mutants	K_m (μmol·L^{-1})	k_{cat} (min^{-1})	k_{cat}/K_m (L·mmol^{-1}·min^{-1})	Mutants	K_m (μmol·L^{-1})	k_{cat} (min^{-1})	k_{cat}/K_m (L·mmol^{-1}·min^{-1})
L21C	94.97	40.76	429.19	G86L	83.86	1.3	15.509	W207N	46.65	13.73	294.32
L21D	68.03	30.29	445.24	G86M	46.02	13.42	291.61	A254I	52.76	16.22	307.43
L21I	125.85	58.58	465.47	M89I	88.71	34.25	386.09	A254M	64.31	8.54	132.79
L21R	83.44	37.27	446.67	M89W	89.72	53.96	601.42	A254R	110.9	3.66	33
L21W	106.6	50.07	469.70	M94E	71.69	7.66	106.85	L256E	57.92	16.28	281.08
M31H	62.3	23.15	371.59	M94F	38.24	12.74	333.16	L256V	61.16	20.14	329.3
M31W	73.716	28.6	387.98	M94K	44.37	6.95	156.64	L256Y	44.39	15.78	355.49
E33D	109.83	46.63	424.57	M94T	43.74	14.25	325.79	F257M	75.22	4.135	54.97
E33N	121.7	51.89	426.38	N95E	100.31	4.84	48.25	F284P	70.54	8.58	121.63
E33T	64.05	18.84	294.15	N95M	101.38	6.3	62.14	V289E	56.15	3.28	58.41
E33V	59.45	21.96	369.387	N95W	73.42	10.28	140.02	V289I	65.61	21.56	328.61
E33W	56.33	28.91	513.23	A156F	69.122	2.134	30.87	V289S	46.66	6.01	128.8
Q36E	51.05	18.64	365.13	A156I	27.896	4.93	176.73	V289N	38.94	2.6	66.77
Q36H	59.28	19.02	320.85	A156V	46.18	11.43	247.51	V289Y	88.96	28.82	323.97
Q36M	59.8	19.92	333.11	I206M	92.78	59.63	642.7	F290W	54.96	23.24	422.85
Q36R	71.19	20.73	291.19	I206N	100.01	73.77	737.63	F290Y	67.68	10.68	157.8
Q36W	74.76	29.26	391.39	W207M	62.95	14.6	231.93	TrL17	65.06	21.56	331.39

Supplementary Table 6 The *Km*, *kcat* and *kcat/Km* value of double and triple substitution variants

Mutants	K_m (μmol·L^{-1})	k_{cat} (min^{-1})	k_{cat}/K_m (L·mmol^{-1}·min^{-1})	Mutants	K_m (μmol·L^{-1})	k_{cat} (min^{-1})	k_{cat}/K_m (L·mmol^{-1}·min^{-1})
L21C/E33N	51.97	20.18	388.3	M89W/I206M	96.08	80.09	833.58
L21C/M89W	54.23	33.13	610.92	M89W/I206N	77.49	71.43	921.8
L21I/E33N	69.16	32.26	466.46	E33N/G86M/L21W	44.73	31.75	709.81
L21I/E33W	75.47	44.84	594.14	E33N/G86M/I206N	39.78	26.56	667.67
L21I/G86M	32.19	21.78	676.61	E33N/M89W/L21I	83.06	46.56	560.56
L21I/M89W	88.69	44.13	497.58	E33N/M89W/L21W	78.22	37.34	477.37
L21I/I206M	85.03	50.03	588.38	E33N/M89W/I206N	102.2	71.94	703.91
L21R/G86M	28.01	13.89	495.89	E33N/I206M/L21I	100.2	65.26	651.3
L21W/E33N	79.35	25.21	317.71	E33N/I206M/L21W	56.36	24.08	427.25
L21W/E33W	76.07	27.84	365.98	E33W/G86M/L21C	19.86	14.62	736.15
L21W/G86M	42.93	30.4	708.13	E33W/G86M/I206M	30.6	21.95	717.32
L21W/M89W	81.87	38.95	475.75	E33W/M89W/L21I	58.71	23.98	408.45
L21W/I206M	100.5	49.1	488.56	E33W/M89W/I206M	76.26	41.55	544.85
L21W/I206N	81.61	59.16	724.91	G86M/M89W/L21W	20.47	7.66	374.21
E33N/G86M	46.41	30.79	663.43	G86M/M89W/E33N	25.38	9.28	365.64
E33N/M89W	64.33	46.4	721.28	G86M/M89W/E33W	26.77	8.89	332.09
E33N/I206M	91.41	53.57	586.04	G86M/M89W/I206M	23.66	14.25	602.28
E33W/G86M	42.66	25.46	596.81	G86M/M89W/I206N	18.45	11.74	636.31
E33W/M89W	62.39	47.05	754.13	M89W/I206M/L21I	67.75	69.1	1019.93
E33W/I206M	91.88	74.17	807.25	M89W/I206N/L21C	82.44	78.37	950.63
G86M/M89W	51.01	9.92	194.47	M89W/I206N/L21I	69.11	73.18	1058.89
G86M/I206N	55.08	36.21	657.41	TrL17	65.06	21.56	331.39
